# Supplementary material for: Comment on ‘YcgC represents a new protein deacetylase family in prokaryotes’
Source: eLife. 2018 Jun 25;7:e37798. doi: 10.7554/eLife.37798 (PMC6023612; doi:10.7554/eLife.37798)
Supplement: Supplementary file 2. [file elife-37798-supp2.docx]

**Supplementary table 2:** Proteases identified in the YcgC sample of Tu et al., which are neither present in the catalytically inactive YcgC S200A preparation from Tu et al. nor in the YcgC preparation from Kremer and Kuhlmann et al.

| **Protein ID** | **Protease** | **Gene name** |
| --- | --- | --- |
| P0A9M0 | Lon protease | lon |
| P0ABC3 | Modulator of FtsH protease HflC | hflC |
| P0ABC7 | Modulator of FtsH protease HflK | hflK |
| P0ABH9 | ATP-dependent Clp protease ATP-binding subunit ClpA | clpA |
| P0AFK0 | Metalloprotease PmbA | pmbA |
| P0AG14 | Probable protease SohB | sohB |
| P25894 | Metalloprotease LoiP | loiP |
| P29745 | Peptidase T | pepT |
| P76403 | Uncharacterized protease YegQ | yegQ |
