# Supplementary material for: Comment on ‘YcgC represents a new protein deacetylase family in prokaryotes’
Source: eLife. 2018 Jun 25;7:e37798. doi: 10.7554/eLife.37798 (PMC6023612; doi:10.7554/eLife.37798)
Supplement: Supplementary file 4. — Usage of the structural alignment programme TM-align to match the first I-TASSER model to all structures in the PDB. The following table shows the top ten proteins that show the closest structural similarity to the predicted I-TASSER model. Due to the structural similarity these proteins often have similar function as the target. [file elife-37798-supp4.docx]

**Supplementary table 4: The YcgC amino acid sequence was analysed by the threading programme I-iTASSER.** Usage of the structural alignment programme TM-align to match the first I-TASSER model to all structures in the PDB. The following table shows the top ten proteins that show the closest structural similarity to the predicted I-TASSER model. Due to the structural similarity these proteins often have similar function as the target.

| **Rank^a^** | | **PDB Hit^b^** | **TM-score^c^** | **RMSD^d^** | **IDEN^e^** | **Cov^f^** |
| --- | --- | --- | --- | --- | --- | --- |
| 1 | | 2xdfA  (PEP-protein phosphotransferase; P08839) | 0.480 | 0.61 | 0.276 | 0.483 |
| 2 | | 1zymA  (PEP-protein phosphotransferase; P08839) | 0.470 | 1.07 | 0.279 | 0.479 |
| 3 | | 2wqdA1  (Beta-ketoacyl-ACP synthase 1; P9WQD9) | 0.449 | 2.36 | 0.226 | 0.487 |
| 4 | | 5t1oB  **(PEP-dependent PTS; P37177)** | 0.429 | 2.69 | 0.218 | 0.477 |
| 5 | | 5t8vA  (uncharacterised protein from *C. thermophilum*; G0S557) | 0.386 | 7.53 | 0.040 | 0.657 |
| 6 | | 2hroA  (PEP-protein phosphotransferase from  *S. carnosus*; P23533) | 0.376 | 3.79 | 0.190 | 0.447 |
| 7 | | 5cskA  (Acetyl-CoA carboxylase from *S. cerevisiae*; Q00955) | 0.373 | 7.48 | 0.057 | 0.623 |
| 8 | | 5h64A  (Ser/Thr kinase mTOR; P42345) | 0.367 | 7.63 | 0.054 | 0.625 |
| 9 | | 2cseU  (Outer capsid protein mu-1; P11077) | 0.364 | 7.26 | 0.062 | 0.600 |
| 10 | | 5ganA  (Pre-mRNA splicing factor 8; P33334) | 0.364 | 6.93 | 0.048 | 0.585 |
| (a) | Ranking of proteins is based on TM-score of the structural alignment between the query structure and known structures in the PDB library | | | | | |
| (b) | PDB Hit is the known structure used for structural alignment with the query structure. | | | | | |
| (c) | TM-score is a metric for measuring the structural similarity of two protein models. | | | | | |
| (d) | RMSD is the RMSD between residues that are structurally aligned by TM-align. | | | | | |
| (e) | IDEN is the percentage sequence identity in the structurally aligned region. | | | | | |
| (f) | Cov represents the coverage of the alignment by TM-align and is equal to the number of structurally aligned residues divided by length of the query protein | | | | | |
