# Supplementary material for: Comment on ‘YcgC represents a new protein deacetylase family in prokaryotes’
Source: eLife. 2018 Jun 25;7:e37798. doi: 10.7554/eLife.37798 (PMC6023612; doi:10.7554/eLife.37798)
Supplement: Supplementary file 5. [file elife-37798-supp5.docx]

**Protein sequences of recombinantly expressed RutR and YcgC proteins from Tu et al:**

**His6 –YcgC; pCA24N**

MRGS**HHHHHH**MVNLVIVSHSSRLGEGVGELARQMLMSDSCKIAIAAGIDDPQNPIGTDAVKVMEAIESVADADHVLVMMDMGSALLSAETALELLAPEIAAKVRLCAAPLVEGTLAATVSAASGADIDKVIFDAMHALEAKREQLGLPSSDTEISDTCPAYDEEARSLAVVIKNRNGLHVRPASRLVYTLSTFNADMLLEKNGKCVTPESINQIALLQVRYNDTLRLIAKGPEAEEALIAFRQLAEDNFGETEEVAPPTLRPVPPVSGKAFYYQPVLCTVQAKSTLTVEEEQDRLRQAIDFTLLDLMTLTAKAEASGLDDIAAIFSGHHTLLDDPELLAAASELLQHEHCTAEYAWQQVLKELSQQYQQLDDEYLQARYIDVDDLLHRTLVHLTQTKEELPQFNSPTILLAENIYPSTVLQLDPAVVKGICLSAGSPVSHSALIARELGIGWICQQGEKLYAIQPEETLTLDVKTQRFNRQGGLCGR

Encoded by pCA24N: MRGS-, GLCGR-; S200: S

**His6 –RutR; pCA24N**

MRGS**HHHHHH**TDPALRATQGAVKTTGKRSRAVSAKKKAILSAALDTFSQFGFHGTRLEQIAELAGVS**K**TNLLYYFPS**K**EALYIAVLRQILDIWLAPLKAFREDFAPLAAIKEYIRLKLEVSRDYPQASRLFCMEMLAGAPLLMDELTGDLKALIDEKSALIAGWVKSGKLAPIDPQHLIFMIWASTQHYADFAPQVEAVTGATLRDEVFFNQTVENVQRIIIEGIRPRGLCGR

Encoded by pCA24N: MRGS, GLCGR, TDPALRA

**YcgC S200A; pET28a N-terminal His_6_-tagged**

MGSS**HHHHHH**SSGLVPRGSHMASMTGGQQMGRGSEFMVNLVIVSHSSRLGEGVGELARQMLMSDSCKIAIAAGIDDPQNPIGTDAVKVMEAIESVADADHVLVMMDMGSALLSAETALELLAPEIAAKVRLCAAPLVEGTLAATVSAASGADIDKVIFDAMHALEAKREQLGLPSSDTEISDTCPAYDEEARSLAVVIKNRNGLHVRPASRLVYTLSTFNADMLLEKNGKCVTPE**A**INQIALLQVRYNDTLRLIAKGPEAEEALIAFRQLAEDNFGETEEVAPPTLRPVPPVSGKAFYYQPVLCTVQAKSTLTVEEEQDRLRQAIDFTLLDLMTLTAKAEASGLDDIAAIFSGHHTLLDDPELLAAASELLQHEHCTAEYAWQQVLKELSQQYQQLDDEYLQARYIDVDDLLHRTLVHLTQTKEELPQFNSPTILLAENIYPSTVLQLDPAVVKGICLSAGSPVSHSALIARELGIGWICQQGEKLYAIQPEETLTLDVKTQRFNRQG

Encoded by pET28a: MGSS**HHHHHH**SSGLVPRGSHMASMTGGQQMGRGSEF; A: S200A

**Proteins sequences of recombinantly expressed RutR and YcgC proteins from our lab:**

**YcgC and YcgC S200A; pGEX-4T5/Tev**

**GGS**MVNLVIVSHSSRLGEGVGELARQMLMSDSCKIAIAAGIDDPQNPIGTDAVKVMEAIESVADADHVLVMMDMGSALLSAETALELLAPEIAAKVRLCAAPLVEGTLAATVSAASGADIDKVIFDAMHALEAKREQLGLPSSDTEISDTCPAYDEEARSLAVVIKNRNGLHVRPASRLVYTLSTFNADMLLEKNGKCVTPE **(S/A)**INQIALLQVRYNDTLRLIAKGPEAEEALIAFRQLAEDNFGETEEVAPPTLRPVPPVSGKAFYYQPVLCTVQAKSTLTVEEEQDRLRQAIDFTLLDLMTLTAKAEASGLDDIAAIFSGHHTLLDDPELLAAASELLQHEHCTAEYAWQQVLKELSQQYQQLDDEYLQARYIDVDDLLHRTLVHLTQTKEELPQFNSPTILLAENIYPSTVLQLDPAVVKGICLSAGSPVSHSALIARELGIGWICQQGEKLYAIQPEETLTLDVKTQRFNRQG

Encoded by pGEX-4T5/TEV: GGS; S200 or S200A: (S/A)

**RutR-His_6_ ; RSF-Duet C-terminally His_6_-tagged**

**MG**MTQGAVKTTGKRSRAVSAKKKAILSAALDTFSQFGFHGTRLEQIAELAGVS**K**TNLLYYFPS**K**EALYIAVLRQILDIWLAPLKAFREDFAPLAAIKEYIRLKLEVSRDYPQASRLFCMEMLAGAPLLMDELTGDLKALIDEKSALIAGWVKSGKLAPIDPQHLIFMIWASTQHYADFAPQVEAVTGATLRDEVFFNQTVENVQRIIIEGIRPR**HHHHHH**

Encoded by pRSF-Duet-1: MG; lysines, which are lysine acetylated: K; His_6_-tag: HHHHHH
